# Supplementary material for: Outcomes of different steroid dosing regimens in critical Covid-19 pneumonia at a Kenyan hospital: A retrospective cohort study
Source: PLoS One. 2024 Aug 20;19(8):e0307265. doi: 10.1371/journal.pone.0307265 (PMC11335146; doi:10.1371/journal.pone.0307265)
Supplement: Attachment — Submitted filename: PLOS ONE response letter - John.docx [file pone.0307265.s003.docx]

**Response to Reviewer’s Comments**

**PLOS ONE**

**OUTCOMES OF DIFFERENT STEROID DOSING REGIMENS IN CRITICAL COVID-19 PNEUMONIA: A RETROSPECTIVE COHORT STUDY**

Thank you for the comments. Please find below the responses to both reviewers’ comments.

**Reviewer 1**

Reviewer #1: Odhiambo J.O. and collaborators performed a single center retrospective observational study to investigate the effect of different posology of corticosteroids (i.e., dexamethasone 6 mg vs. higher equivalent dosages) in patients suffering from critical COVID-19 pneumonia admitted to ICU of the Aga Khan Hospital in Nairobi, Kenya. I personally worked in a European COVID ICU during the pandemic, and it was very complicated and stressful. I imagine working in a low and middle-income country ICU in such a situation could be exhausting; thus, I would like to thank you for conducting this research effort, and I hope this review may help you.

The primary outcome of the study was mortality. Secondary outcomes were the occurrence of superinfections, dysglycemia, and duration of ICU length of stay.

Two-hundred-eighty-eight patients entered the analysis. 48% received dexamethasone 6 mg; 52% higher dosages. Overall, 45.5% of patients died. No difference in mortality was observed in the two groups (43.7% vs. 47.9%, p=0.549). Higher corticosteroid dosages seemed to be associated with a higher incidence of dysglycemia and superinfection events.

The authors have provided a description of an extensively studied topic but considered a population rarely enrolled in trials and studies. Indeed, the major strength of this work is the completely African population included. This aspect, in my opinion should be underlined, both in the title, as well as in the abstract.

Response: We have revised and added “At a Kenyan Hospital” in the title.

Nevertheless, the manuscript, in its current form, requires some improvements and presents a lot of methodological issues and limitations that should be thoroughly addressed:

- Follow STROBE guidelines to report observational study results, as required by the Journal publication criteria.

Response: Thank you. We have attached the STROBE Checklist and submitted as per the criteria.

- Please specify better the primary outcome: did you assess ICU mortality, hospital mortality, or 28-day mortality? Moreover, I would suggest adding the primary outcome result to the main text as well as in the table.

Response: The primary outcome was ICU Mortality and we have edited the manuscript accordingly.

- Please, it could be interesting and could improve the quality of the manuscript to report 1) at least a score or parameters of illness severity (i.e., P/F ratio, Respiratory Rate, SOFA or SAPS score…); 2) the number of patients who needed mechanical ventilation; 3) the kind of steroid administered; 4) the timing of corticosteroid therapy starting compared to ICU admission.

Response: I agree, this could improve the quality of the study. However, we did not collect data on the different scoring systems. Additionally, we only included the steroid doses in dexamethasone equivalence but not the specific steroids used.

- Univariate statistic tests used are completely described, but I would suggest adjusting for possible confounders (i.e., age, sex, comorbidities, the severity of illness) for a better evaluation of steroid dosage impact on outcome. Moreover, if data permits, try to perform a survival analysis (and try to construct a Kaplan-Meier curve figure).

Response: Unfortunately, we did not collect survival data and thus it was beyond the scope of this study to do Kaplan Meier and survival analysis.

- I would suggest reporting outcome measures in the outcome results section, not in the population characteristic section.

Response: We have tried to edit the manuscript accordingly.

- In the limitation section of the discussion, you stated that missing data may have influenced the results. Please report the number of missing data for each variable.

Response: Thank you. We have included this in the limitation section. Only five patients were excluded from the study due to incomplete records.

- I would suggest reporting Table 1, joined with Table 2, and reporting the percentage of subjects in Table 2 columns.

Response: We have retained the tables as it is on the flow of the results.

**Reviewer 2**

Reviewer #2: The study is good but, being a retrospective study, it has its own limitations as has been pointed by the authors. A few questions for the authors-

1. They have mentioned high dose of steroids as any dose more than 6 mg dexamethasone, were patients on any other steroids like prednisone, methylprednisolone? When were the steroids started in these patients, as in within how many days of onset of symptoms?

Response: Yes, other types of steroids were used but in the data collection tool we only included steroid doses which were all converted to dexamethasone into dexamethasone equivalence.

2. Was there any comparison between the survivors and non survivors in terms of oxygen requirements, ventilators-NIV or invasive?

Response: Thank you for this. This may have been a good comparison to do. All the patients included in the study were on ventilatory support, but we did not include the specific type of support.

3. Was there any correlation between the d Dimer levels amongst the survivors and non survivor groups?

Response: No data was collected on D-dimer levels or any other coagulation parameters.

4. How many patients were on Remdesevir considering that these were the more severe cases and was there any correlation on this?

Response: This was beyond the scope of our study and has been included as part of the limitations.

5. How many of these patients also received LMWH or oral anticoagulants and did this affect the outcome of the study?

Response: We did not include data on the anticoagulation. However, as per the hospital policy and guidelines, all patients admitted to critical care unit are put on prophylactic anticoagulation or treatment doses in case of evidence of thrombosis unless there are contraindications.

6. How many patients underwent a bronchoscopy and BAL for the diagnosis of pneumonia?

Response: Thank you for this. Unfortunately, this was beyond the scope of our study.
