# Supplementary material for: Outcomes of different steroid dosing regimens in critical Covid-19 pneumonia at a Kenyan hospital: A retrospective cohort study
Source: PLoS One. 2024 Aug 20;19(8):e0307265. doi: 10.1371/journal.pone.0307265 (PMC11335146; doi:10.1371/journal.pone.0307265)
Supplement: Attachment — Submitted filename: PLOS ONE response letter - 8th April.docx [file pone.0307265.s004.docx]

**Response to Reviewer’s Comments**

**PLOS ONE**

**OUTCOMES OF DIFFERENT STEROID DOSING REGIMENS IN CRITICAL COVID-19 PNEUMONIA: A RETROSPECTIVE COHORT STUDY**

Thank you for the comments. Please find below the responses to both reviewers’ comments.

Reviewer #1: Unfortunately, the authors, despite the changes in the manuscript were not able to address any of the instance I raised.

Response: We appreciate the comments and suggestions from Reviewer 1. Some of the suggestions such as reporting at least a score or parameters of illness severity (i.e., P/F ratio, Respiratory Rate, SOFA or SAPS score…) or performing survival analysis was beyond the scope of the current project as the different data was not collected. Furthermore, we attached the STROBE guidelines to report the observational study as requested. We tried to address as much as possible and edited the manuscript accordingly.

Reviewer #2: All comments have been addressed to satisfaction and the article can be accepted if found to be suitable for publication by the editor.

Response: We appreciate the comments and suggestions from Reviewer 2.

Reviewer #3: It is difficult to conclude that high doses of steroids do not influence the prognosis. Can you compare the data that can assess the severity of the 2 groups? Particularly saturation at admission and during progression or add this mention to the limitations of this study.

Response: We appreciate the comments from Reviewer 3. We included all the patients with critical COVID-19 and on ventilator support as per the inclusion criteria. However, we did not collect any data on the measures of severity such as oxygen saturations. We have, therefore, added it to our limitations as you have advised.
