# Supplementary material for: Outcomes of different steroid dosing regimens in critical Covid-19 pneumonia at a Kenyan hospital: A retrospective cohort study
Source: PLoS One. 2024 Aug 20;19(8):e0307265. doi: 10.1371/journal.pone.0307265 (PMC11335146; doi:10.1371/journal.pone.0307265)
Supplement: Attachment — Submitted filename: PLOS ONE Comments- John 14th June 2024.docx [file pone.0307265.s005.docx]

**Response to Reviewer’s Comments**

**PLOS ONE**

**OUTCOMES OF DIFFERENT STEROID DOSING REGIMENS IN CRITICAL COVID-19 PNEUMONIA AT A KENYAN HOSPITAL: A RETROSPECTIVE COHORT STUDY**

**If the authors have adequately addressed your comments raised in a previous round of review and you feel that this manuscript is now acceptable for publication, you may indicate that here to bypass the “Comments to the Author” section, enter your conflict of interest statement in the “Confidential to Editor” section, and submit your "Accept" recommendation.**

**Reviewer #1: (No Response)**

**Reviewer #2: All comments have been addressed**

**Reviewer #3: All comments have been addressed**

**Reviewer #4: All comments have been addressed**

*Response: Thank you for taking time out of your schedule to review our paper and we are happy to note that all your queries were addressed.*

**Reviewer #1:** The choice not to collect further descriptive data from the two cohorts, unfortunately, is not agreeable. The severity data of the pathology wouldn't have had solely a descriptive purpose but could have helped the authors to consider possible confounders in the statistical analysis: such as whether patients who had taken higher doses of steroids were the most severe and thus at higher risk of superinfection

*Response: Having realised that this would improve the quality of the manuscript as was highlighted by the reviewer, we went back and collected data on markers of severity of the disease for instance CRP and oxygen saturation at admission. Due to medical records being paper documents and also limited data available, we aimed at collecting CRP and oxygen saturation. From furtner analyses, it was evident that oxygen saturation at admission was significantly lower in the non-survivors’ group than the survivors’ group whereas as CRP was significantly lower in the high dose group as compared to the standard dose group.*

**Reviewer #2:** All queries have been addressed to satisfaction and may be considered for publication if the article meets the other requirements.

*Response: Thank you for taking time out of your schedule to review our paper and we are happy to note that all your queries were addressed.*

**Reviewer #4:** The points mentioned by the authors in the limitation are very important for the study. The discussion section is inadequate

*Response: Thank you so much for the point you have raised. We have delt with one of the major limitations which was lack of data on the markers of severity such as the level of the inflammatory markers and oxygen saturations at admission and put up an additional paragraph in the discussion section. We believe that this has improved the quality of the manuscript and hopefully accepted for publication.*
